# The impact of neglecting feature scaling in k-means clustering

**DOI:** 10.1371/journal.pone.0310839

**Published:** 2024-12-06

**Authors:** Chantha Wongoutong

**Affiliations:** Department of Statistics, Faculty of Science, Kasetsart University, Bangkok, Thailand; Mae Fah Luang University, THAILAND

## Abstract

Despite the popularity of *k-*means clustering, feature scaling before applying it can be an essential yet often neglected step. In this study, feature scaling via five methods: Z-score, Min-Max normalization, Percentile transformation, Maximum absolute scaling, or RobustScaler beforehand was compared with using the raw (i.e., non-scaled) data to analyze datasets having features with different or the same units via *k-*means clustering. The results of an experimental study show that, for features with different units, scaling them before *k-*means clustering provided better accuracy, precision, recall, and F-score values than when using the raw data. Meanwhile, when features in the dataset had the same unit, scaling them beforehand provided similar results to using the raw data. Thus, scaling the features beforehand is a very important step for datasets with different units, which improves the clustering results and accuracy. Of the five feature-scaling methods used in the dataset with different units, Z-score standardization and Percentile transformation provided similar performances that were superior to the other or using the raw data. While Maximum absolute scaling, slightly more performances than the other scaling methods and raw data when the dataset contains features with the same unit, the improvement was not significant.

## 1. Introduction

*K-*means clustering is a distance-based algorithm used to group or cluster items according to measured or perceived intrinsic characteristics or similarities [1]. This technique used to discover natural grouping(s) of patterns, points, or objects is invaluable for analyzing large databases of multivariate data involving many features used in the fields of data mining [2], statistical data analysis [3], pattern recognition [4], and image processing [5].

Initialization typically influences the *k-*means clustering algorithm and must be provided with the number of clusters beforehand [6]. In general, initial cluster centers are chosen randomly, which affects the final cluster formation; this implies that the clustering outcome can differ each time the algorithm is executed, even on the same dataset. Many researchers have proposed initial cluster centers for the *k-*means algorithm [7–10]. Although it is essential to determine the number of clusters required for *k-*means clustering, this can be challenging for end-users who do not have an in-depth understanding of the dataset. Several methods have been proposed in the literature to address this issue, including the rule of thumb [11], the elbow method [12], the information criterion approach [13], the information theoretical approach [14], choosing the number of clusters (*k*) using the silhouette method [15], and cross-validation [16].

However, the *k*-means technique requires feature scaling in the pre-processing stage, a process that is often overlooked. The *k-*means algorithm relies on distance-based metrics (e.g., the Euclidean distance) to mitigate scale variation. This means the results can vary depending on the range of values used for the features, and the smaller the distance, the closer the points will be to each other, thereby indicating their similarity [17]. Especially when analyzing real-world datasets, the features can have different scales because they are measured using different units. Scaling the features into a uniform range to avoid any feature becoming predominant in the distance calculation and to help improve the clustering results is crucial to enhance the *k-*means algorithm’s performance [18, 19]. Patel and Mehta [20] claimed that a normalized dataset produces better outcomes during the raw data clustering process. According to recent research [21] a high magnitude affects the distance between two given points, which impacts the performance of *k-*means clustering since variables with higher magnitudes are given more weight. Hence, it is always advisable to bring all the features to the same scale for applying distance-based algorithms such as *k-*means clustering [22].

Although feature scaling is commonly recommended as a pre-processing step before clustering, there is limited research on how the scaling of features affects the clustering performance, especially when all of the features have the same or different units. Therefore, the aim of the present study is to investigate and clarify whether feature scaling is essential as a pre-processing step by examining two scenarios: datasets in which the units of the features are the same or different.

The remainder of this paper is organized as follows. In section 2, datasets used in the study are presented, while Section 3 provides the methodology for *k-*means clustering and feature scaling. The performance metrics and experimental study are covered in Section 4, the results of which and a discussion thereon are provided in Section 5. Finally, conclusions on the study are imparted in Section 6.

## 2. The datasets used in the study

To examine the impact of the prior feature scaling for *k-*means clustering, 10 real-world datasets were obtained from [23] https://www.kaggle.com/, the details of which are provided in Table 1.

**Table 1:** The datasets used in this research.

Data	Description	No. of Features	Units of Features	No. of Objects	No. of Classes	No. of Object in Each Class
D1	Penguins	4	Different	342	3	151,68,123
D2	Wine quality	13	Different	178	3	59,71,48
D3	Breast cancer	30	Different	569	2	357, 212
D4	Low-density lipoprotein	3	Different	569	3	56, 214, 299
D5	Diamonds	4	Different	351	2	200,151
S1	Bacteria strains	5	Same	272	3	143,77,52
S2	Iris	4	Same	150	3	50,50,50
S3	Italian olives	8	Same	572	3	151,98,323
S4	Crabs	5	Same	200	2	100,100
S5	Rainfall	5	Same	108	3	8,78,22

## 3. Methodology for *k-*means clustering and feature scaling

### 3.1 *K-*means clustering

Data clustering is a crucial technique for many applications such as data mining and is a valuable tool for researchers working with large datasets of multivariate data [24]. Several clustering methods are available, each with its inherent advantages and disadvantages. *K-*means, a centroid-based clustering algorithm that is the most well-known data mining method, is a simple yet powerful clustering technique [25, 26]. In this method, an iterative algorithm is used to partition the dataset into *k* distinct non-overlapping subgroups (clusters), where each data point belongs to only one group [27]. Besides, the inter-cluster data points are simultaneously made as similar as possible while keeping the different clusters as far apart as possible. The working of *k-*means clustering is explained in the following steps:

1. Select a suitable value for *k*.
2. Determine the initial centroids by randomly assigning them from the existing data until *k* equals the number of initial centroids.
3. Assign each data point to its closest centroid, forming the predefined value of *k*.
4. Calculate the distance and place a new centroid in each cluster.
5. Repeat the third step, which means reassigning each data point to the new closest centroid in each cluster.
6. If any reassignment occurs, go to step 4; otherwise, finish.

**Fig 1:**
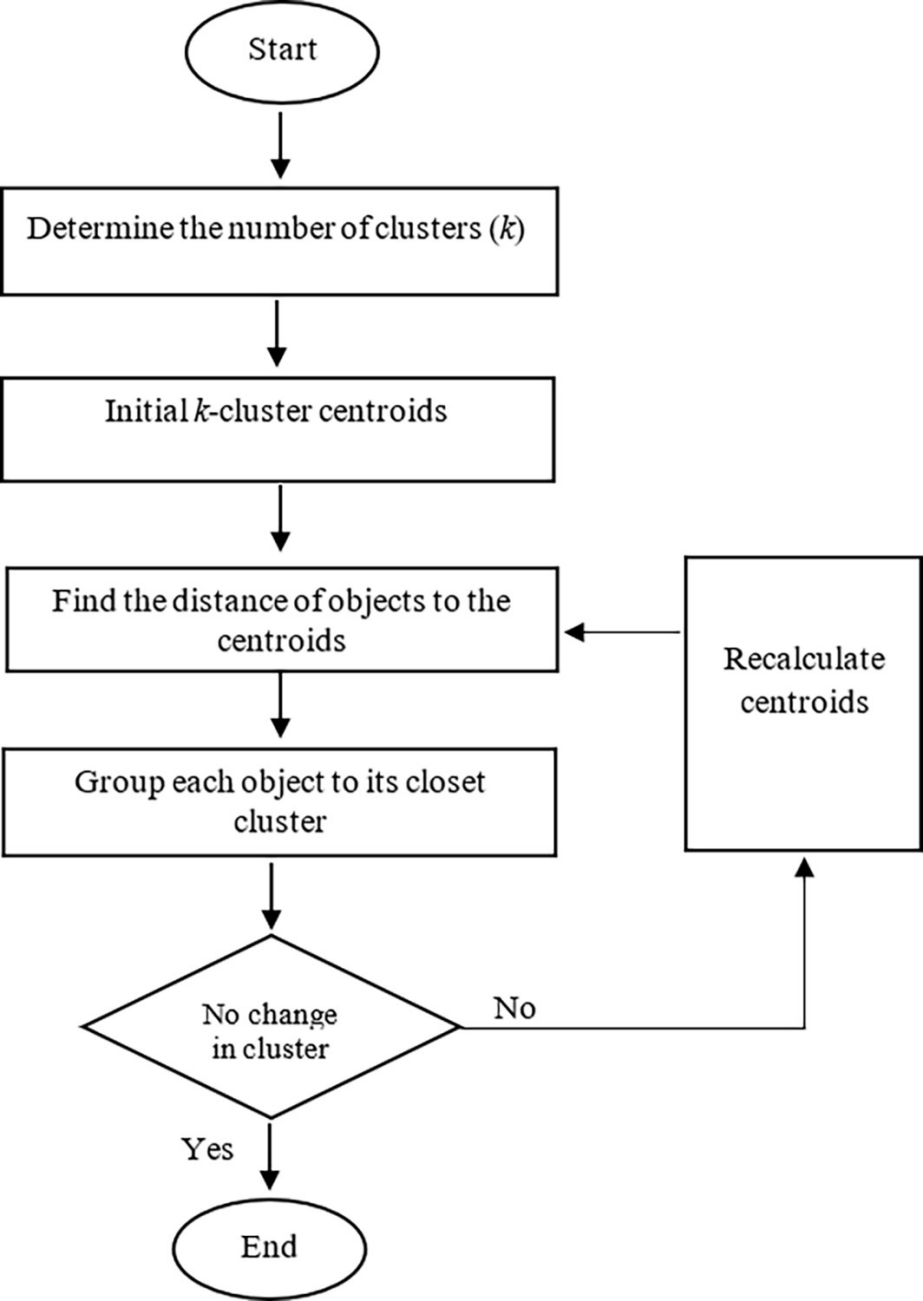
A flowchart for *k-*means clustering.

A flowchart for the *k-*means clustering algorithm is illustrated in Fig 1.

### 3.2 The feature scaling methods

Feature scaling is a crucial step in the pre-processing stage of machine-learning when real-world datasets have many features with a wide range of values. Hence, transforming a dataset’s variables (features) from different dynamic ranges to a specific range and ensuring that no single feature predominates in the distance calculations for the algorithm can help improve its performance [28]. The most common techniques of feature scaling consist of normalization and standardization [29]. Normalization is used when there is a need to bind the values between two numbers, typically between [0,1], while standardization transforms the data to zero mean and unit variance, thereby making it unitless. Some machine-learning algorithms, such as *k-*means clustering, are most affected by the range of features. Behind the scenes, *k-*means clustering uses distances (usually the Euclidean distance) between data points to determine their similarity. Hence, before using *k-*means clustering, feature scaling is crucial [30, 31]. In the present study, five scaling methods were used: Min-Max normalization, Z-score, Percentile transformation, and Maximum absolute scaling and RobustScaler.

#### 3.2.1. Z-score

This is a standard scoring method since the distribution of the scores on the various variables is standardized. This technique scales the values of a feature with zero mean and unit variance [32]. It is calculated by subtracting the mean of the feature from each value and then dividing it by the standard deviation. The Z-score values can be positive or negative: negative and positive signs represent an observation below or above the mean, respectively. The equation for calculating the Z-score is

$$x^{\prime }=\frac{x-\bar{x}}{S}.$$ (1)

#### 3.2.2. Min-max normalization

In this method, the raw data are scaled within a specific range [0,**1**] while preserving the relationships among them [33]. It is calculated by subtracting the minimum value from each feature value and then dividing it by the range of that feature. Thereby, the standard deviation of the data scale has a smaller value. Moreover, the effect of outliers is suppressed to a certain extent. The equation to achieve this is

$$x^{\prime }=\frac{x-\min(x)}{\max(x)-\min(x)}.$$ (2)

#### 3.2.3. Percentile transformation

The percentile is used to compare the values in the given data or to find where the dataset’s value stands when compared to other candidates [34]. The benefit of the percentile is that each value has a relatively straightforward interpretation; it is the percentage of observations with that value or below it. It is simple to calculate by considering the percentile of *x*, which is the ratio of the number of values below (*n*) *x* to the total number of values (*N*) multiplied by 100; i.e.,

$$x^{\prime }=\frac{n}{N}\times 100.$$ (3)

#### 3.2.4 Maximum absolute scaling

aims to bring different features or variables onto a common scale, thereby enabling a fair comparison and improving the performance of machine-learning algorithms. Maximum absolute scaling is one of the most commonly used methods for normalization. It is computed by dividing each observation by the maximum absolute value of the variable. Hence, the preceding transformation results in a distribution in which the values vary approximately within the range of -1 to 1. The equation for Maximum absolute scaling is

$$x^{\prime }=\frac{x}{\max(|x|)}.$$ (4)

#### 3.2.5. RobustScaler

RobustScaler is a scaling method that uses the median. It removes the median and scales the data based on the interquartile range (IQR), which ranges between the 25th and 75th percentile. Because it uses the interquartile range, it can handle outliers well while scaling the data. The equation to achieve this is

$$x^{\prime }=\frac{x-x_{median}}{IQR}.$$ (5)

## 4. The performance metrics and experimental study

The different feature scaling methods and using the raw data on the performance of *k-*means clustering. Two scenarios were considered: datasets with the same or different units and five scaling methods were used: Z-score, Min-Max normalization, Percentile transformation, Maximum absolute scaling, and RobustScaler.

### 4.1 The performance metrics

Evaluating the performance of *k-*means clustering is impossible when the true data grouping is unknown. Therefore, datasets in which the true group membership is known were obtained, and different metrics were used to evaluate the performances of the feature scaling methods. A confusion matrix was created from the true grouped data and *k-*means clustering results and used to compare the accuracy, precision, recall, and F-scores of the methods. Thereby, the number of misclassified patterns and the total number of patterns for each dataset were evaluated.

#### 4.1.1 Confusion matrix

A confusion matrix is a popular measure used to solve classification problems; it summarizes the predictions made by a classification model organized into a table by class [35]. Also, it helps gain insight into how correct the predictions were and how they hold up against the actual values. Table 2 provides an example of binary classification using a confusion matrix of predicted and actual values.

**Table 2:** Binary classification using a confusion matrix.

Predicted Values		Actual Values	
		Positive	Negative
	Positive	True positive	False positive
	Negative	False negative	True negative

#### 4.1.2 Accuracy

Accuracy is one of the most frequently used metrics for evaluating classifier models. To calculate accuracy, counts of correctly classified observations are divided by the total number of observations, which is easy to understand and implement [36]. While accuracy proves to be one of the most popular classification metrics because of its simplicity, it has a few major shortcomings, such as an imbalanced dataset. The equation for accuracy is

$$Accuracy=\frac{TP+TN}{TP+TN+FP+FN},$$ (6)

where TP, TN, FP, and FN are the true positive, true negative, false positive, and false negative values, respectively.

#### 4.1.3 Precision and recall

Precision and recall are two metrics that can help differentiate between error types and can still prove helpful for problems in scenarios with class imbalance [37]. The respective equations are

Precision=TPTP+FPandRecall=TPTP+FN. (7)

#### 4.1.4 F-score

The F-score is another valuable metric, especially for imbalanced datasets. It balances precision and recall, thereby more comprehensively evaluating a model’s performance. It is beneficial when there is a need to assess both false positives and false negatives by offering a single numerical representation that considers both aspects of classification accuracy [38]. The equation to achieve this is

Fscore=2×(Precision×Recall)(Precision+Recall). (8)

### 4.2 Experimental study

**Fig 2:**
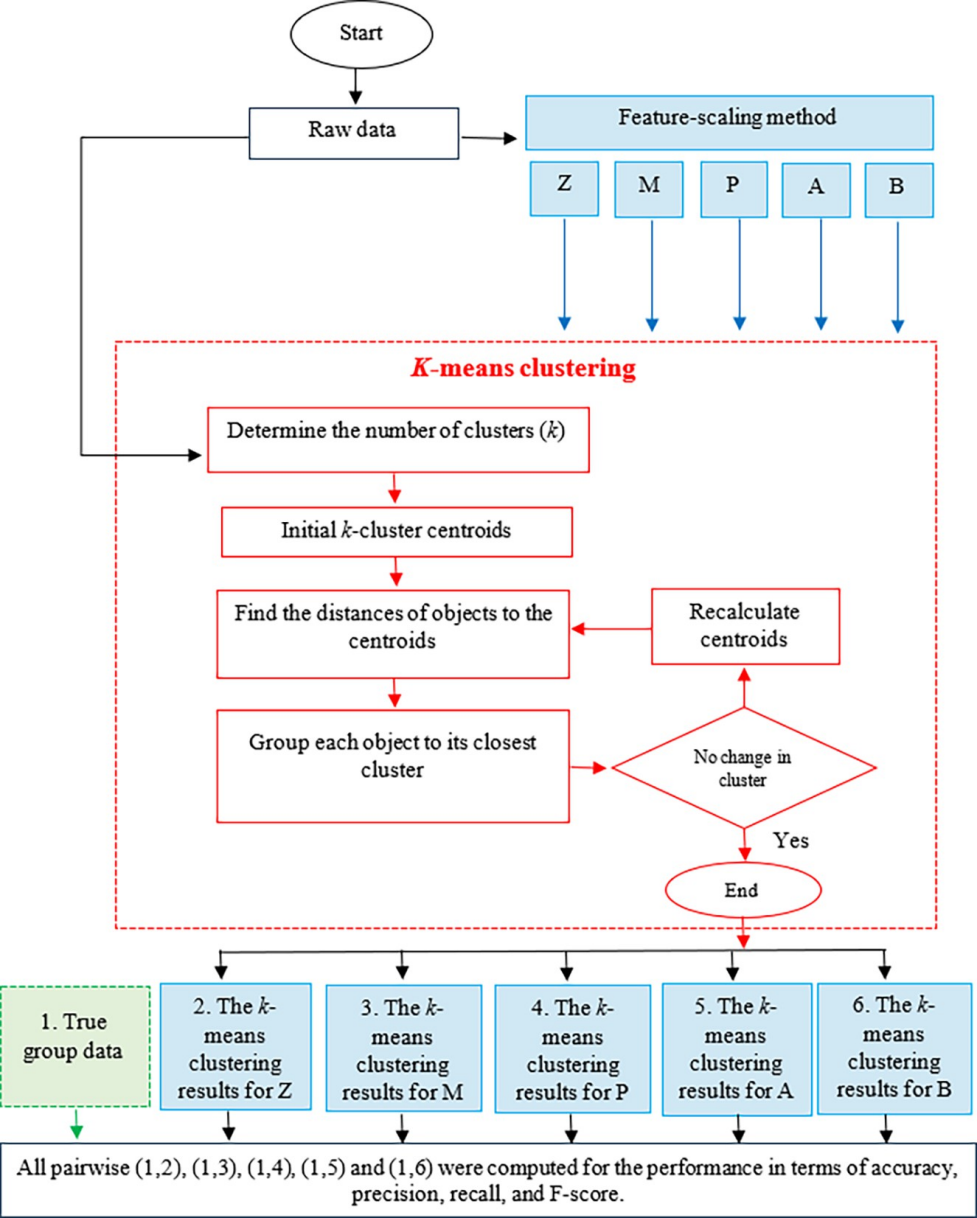
A flowchart for feature scaling by using Z: Z-score, M: Min-Max normalization, P: Percentile transformation, and A: Maximum absolute scaling and B: RobustScaler and the *k-*means clustering results.

Ten datasets were obtained for the study: five with the same unit for all of the features and five with different units. The five scaling methods: Z-score, Min-Max normalization, Percentile transformation, Maximum absolute scaling, and RobustScaler were used for each of the datasets. *k-*means clustering for each dataset was computed using the raw data and after applying the five scaling methods. Performance in terms of accuracy, precision, recall, and F-score was calculated by comparing the true grouped data and the results of the *k-*means clustering. A flowchart of the overall process is presented in Fig 2.

## 5. Results and discussion

### 5.1. Heatmaps for data visualization

Visualization is simple and valuable for exploring the overall structure of results. One of the most popular graphical methods for visualizing high-dimensional data is the heatmap, in which a table of numbers is encoded as a grid of colored cells; it can be used to explore in-depth and effective information based on the results. The arrangement of rows and columns in a heatmap matrix is ordered to accentuate patterns, often complemented by dendrograms. Heatmaps are used in various forms of analytics for visualizing, including observations, correlations, patterns of missing values, and more. Their versatility makes them a valuable tool for distilling complex information into visually accessible representations.

**Fig 3:**
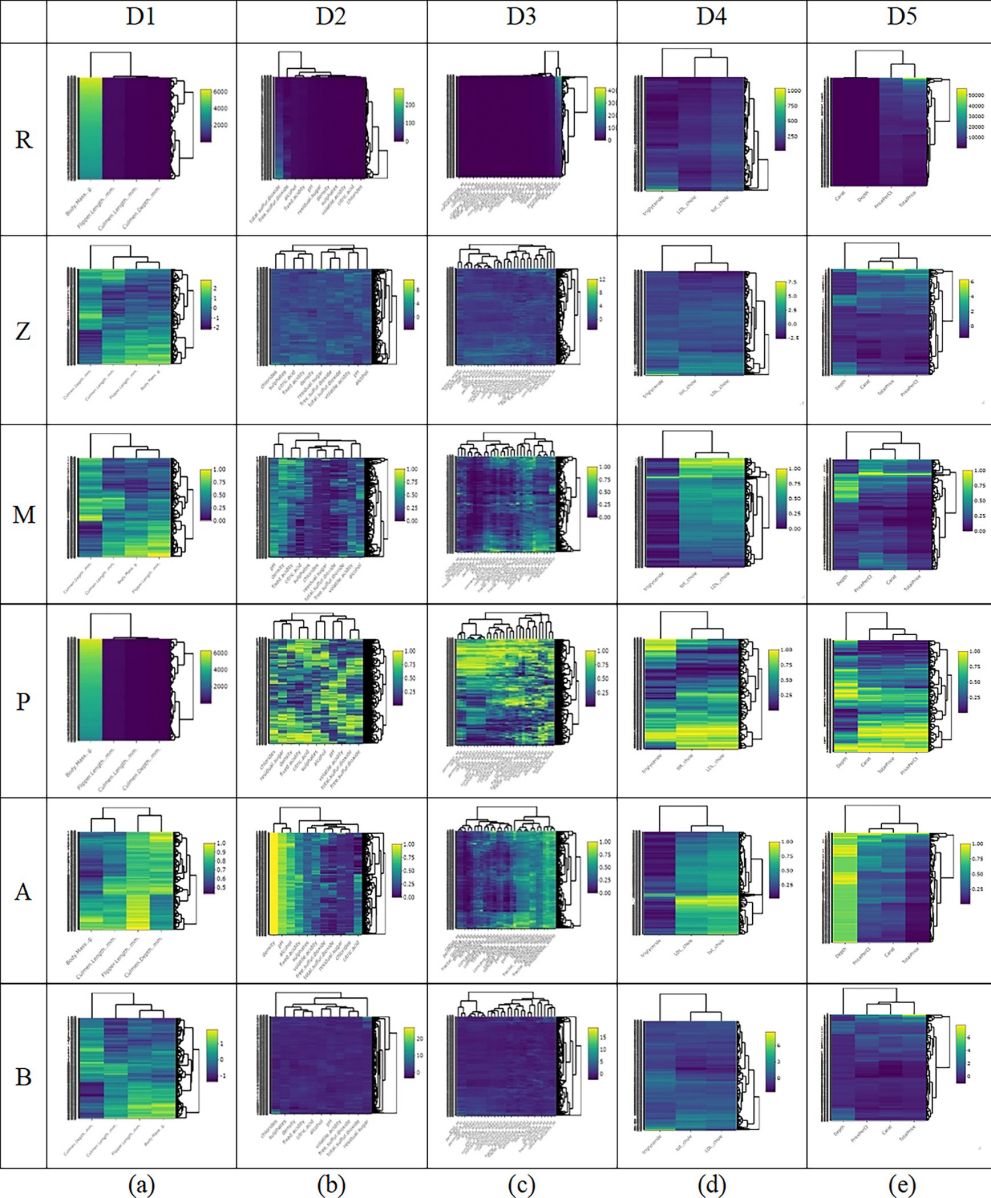
Heatmaps for datasets (a) D1, (b) D2, (c) D3, (d) D4, and (e) D5. R: raw data, Z: Z-score, M: Min-Max normalization, P: Percentile transformation, A: Maximum absolute scaling and B: RobustScaler.

Fig 3 shows a heatmap for the results using the five datasets in which the features in each dataset have different units (D1–D5). For example, D1(R) is a raw dataset of three penguin species with four features: culmen length (mm), culmen depth (mm), flipper length (mm), and body mass (g). Obviously, a pattern could not be detected in the plotted heatmap when using the raw data because the unit for body mass (grams) is different from the other three features (mm), which impacts the Euclidean distance used to measure the similarity.

Feature scaling using Z-score is shown in Fig 3 as heatmap D1 (Z); some object patterns can be seen in the structure of this data as a dendrogram to the right of the heatmap. Heatmap D1 (M) is after normalizing features to [0,**1**] using Min-Max normalization. We can see that the heatmap plot of this dataset represents a pattern that is easier to explore when compared with the heatmap of raw data. Furthermore, scaling via Percentile transformation and normalizing all features to [0,**1**] resulted in heatmap D1 (P), in which the pattern is easier to explore when compared with the heatmap using raw data. Likewise, scaling data by using Maximum absolute scaling resulted in heatmap D1 (A), in which all features are normalized to [0,**1**]. Last, scaling data using RobustScaler resulted in heatmap D1 (B), similar to Z-score but using the interquartile range; it can handle outliers well while scaling the data.

Moreover, compared with the heatmap using raw data, we can see that the dendrogram uncovers some object patterns. When there were more than 10 features, such as D2 and D3, it was difficult to gain insights from the heatmaps using the raw datasets because of the different units of the features; the shade of color and the arrangement of the rows in the dendrogram is not conducive to detecting the classifier pattern.

**Fig 4:**
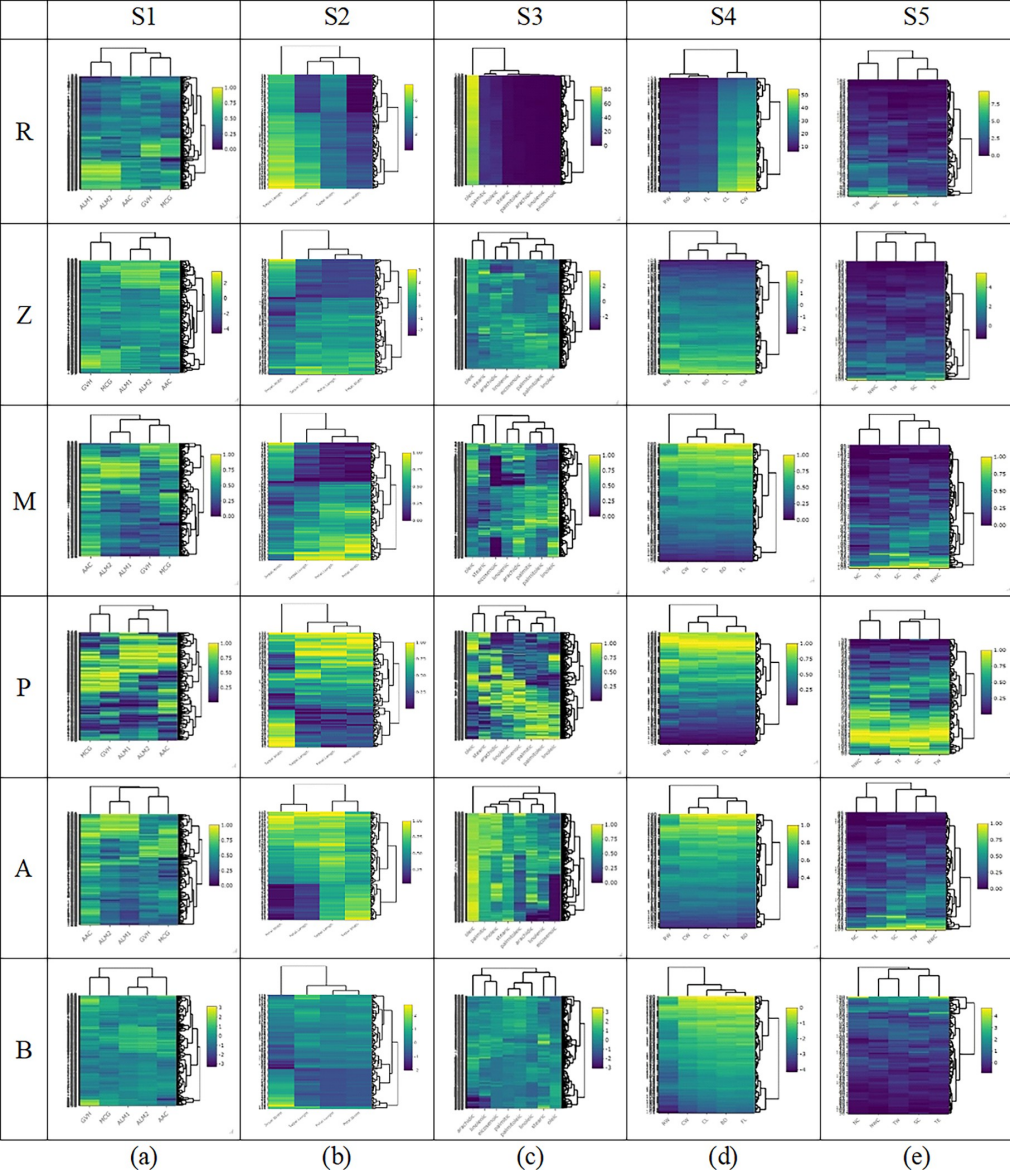
Heatmaps for datasets (a) S1, (b) S2, (c) S3, (d) S4, and (e) S5. R: raw data, Z: Z-score, M: Min-Max normalization, P: Percentile transformation, A: Maximum absolute scaling and B: RobustScaler.

On the other hand, Fig 4 illustrates heatmaps for the five datasets in which the features in each dataset have the same unit (S1–S5). Heatmap visualization using the raw data and the five scaling methods provide easily explorable patterns. Thus, feature scaling is essential before *k-*means clustering when a dataset has features with different units.

### 5.2 Performance evaluation of feature scaling before *k-*means clustering

The results for performance metrics accuracy, precision, recall, and F-score and also testing the hypothesis for homogeneity between the true grouped data and the results of *k-*means clustering for the datasets with different and the same unit are reported in Tables 3 and 4, respectively.

**Table 3:** The performance results for *k-*means clustering and testing the hypothesis for homogeneity between the true grouped data and feature scaling on datasets containing features with different units.

Data	Feature-Scaling Method	Accuracy	Precision	Recall	F-Score	Chi-square Value (p-Value)
D1	None	0.5614	0.4949	0.6058	0.5448	19.829 (<0.001)**
	Z-score	0.9620	0.9578	0.9512	0.9545	0.157 (0.925)^ns^
	Min-Max normalization	0.8538	0.8360	0.8395	0.8378	4.014 (0.134)^ns^
	Percentile	0.8889	0.8973	0.8693	0.8831	1.697 (0.428)^ns^
	Maximum absolute scaling	0.9152	0.8951	0.8978	0.8965	1.142 (0.565)^ns^
	RobustScaler	0.9327	0.9333	0.9297	0.9315	1.192 (0.551)^ns^
D2	None	0.7022	0.6960	0.7237	0.7096	0.695 (0.706)^ns^
	Z-score	0.9663	0.9718	0.9643	0.9680	0.162 (0.922)^ns^
	Min-Max normalization	0.9494	0.9577	0.9496	0.9537	0.376 (0.829)^ns^
	Percentile	0.9775	0.9812	0.9748	0.9780	0.071 (0.965)^ns^
	Maximum absolute scaling	0.9157	0.9273	0.9219	0.9246	0.885 (0.642)^ns^
	RobustScaler	0.9607	0.9662	0.9617	0.9639	0.133 (0.936)^ns^
D3	None	0.8541	0.8052	0.9026	0.8511	13.318 (<0.001)**
	Z-score	0.9104	0.8931	0.9143	0.9036	1.429 (0.232)^ns^
	Min-Max normalization	0.9279	0.9119	0.9341	0.9229	0.162 (0.687)^ns^
	Percentile	0.9051	0.9110	0.8948	0.9028	1.197 (0.274)^ns^
	Maximum absolute scaling	0.9209	0.9044	0.9262	0.9152	0.162 (0.687)^ns^
	RobustScaler	0.9156	0.8925	0.9295	0.9107	2.560 (0.110)^ns^
D4	None	0.5202	0.4405	0.5025	0.4695	56.953 (<0.001)**
	Z-score	0.8348	0.8704	0.8067	0.8373	2.568 (0.277)^ns^
	Min-Max normalization	0.8190	0.8590	0.7901	0.8232	2.705 (0.259)^ns^
	Percentile	0.8366	0.8724	0.8047	0.8372	2.260 (0.323)^ns^
	Maximum absolute scaling	0.8207	0.8588	0.7968	0.8267	3.365 (0.186)^ns^
	RobustScaler	0.8172	0.8597	0.7607	0.8072	2.044 (0.360)^ns^
D5	None	0.6724	0.6192	0.8175	0.7047	53.358 (<0.001)**
	Z-score	0.8974	0.8816	0.9209	0.9008	2.657 (0.103)^ns^
	Min-Max normalization	0.8775	0.8650	0.9119	0.8878	1.749 (0.186)^ns^
	Percentile	0.9316	0.9392	0.9307	0.9349	0.558 (0.1455)^ns^
	Maximum absolute scaling	0.8832	0.8650	0.9119	0.8878	3.589 (0.058)^ns^
	RobustScaler	0.8974	0.8930	0.8973	0.8951	0.209 (0.648)^ns^

Note

*, **, and ^ns^ represent significant differences at the 0.05 and 0.01 levels and non-significance, respectively.

**Table 4:** The performance results for *k-*means clustering and testing the hypothesis for homogeneity between the true grouped data and feature scaling on datasets containing features with the same unit.

Data	Feature-Scaling Method	Accuracy	Precision	Recall	F-Score	Chi-Square Value (p-Value)
S1	None	0.9191	0.9164	0.9073	0.9119	0.034 (0.983)^ns^
	Z-score	0.9338	0.9257	0.9276	0.9267	0.111 (0.946)^ns^
	Min-Max normalization	0.9228	0.9187	0.9122	0.9154	0.048 (0.976)^ns^
	Percentile	0.9375	0.9240	0.9338	0.9289	0.199 (0.905)^ns^
	Maximum absolute scaling	0.9191	0.9164	0.9073	0.9119	0.034 (0.983)^ns^
	RobustScaler	0.9228	0.9218	0.9223	0.9220	0.144 (0.946)^ns^
S2	None	0.8933	0.8933	0.9072	0.9002	1.428 (0.490)^ns^
	Z-score	0.8533	0.8533	0.8647	0.8590	2.027 (0.363)^ns^
	Min-Max normalization	0.8867	0.8867	0.8979	0.8922	1.360 (0.507)^ns^
	Percentile	0.8533	0.8533	0.8548	0.8541	0.744 (0.689)^ns^
	Maximum absolute scaling	0.9600	0.9600	0.9605	0.9602	0.085 (0.958)^ns^
	RobustScaler	0.9067	0.9067	0.9132	0.9099	0.595 (0.743)^ns^
S3	None	0.6993	0.7504	0.6902	0.7190	8.085 (0.018)*
	Z-score	0.7010	0.7354	0.7881	0.7608	11.885(0.003)**
	Min-Max normalization	0.7675	0.8298	0.8081	0.8188	5.828 (0.054)^ns^
	Percentile	0.7587	0.7973	0.7572	0.7768	4.126 (0.127)^ns^
	Maximum absolute scaling	0.7710	0.8353	0.8198	0.8275	6.845 (0.033)*
	RobustScaler	0.7605	0.8526	0.7412	0.7930	7.194 (0.027)*
S4	None	0.5900	0.5900	0.5900	0.5900	0.085 (0.770) ^ns^
	Z-score	0.6100	0.6110	0.6100	0.6100	0.326 (0.568) ^ns^
	Min-Max normalization	0.6100	0.6120	0.6110	0.6100	0.735 (0.391) ^ns^
	Percentile	0.6200	0.6200	0.6200	0.6200	0.179 (0.672) ^ns^
	Maximum absolute scaling	0.6100	0.6120	0.6110	0.6100	0.735 (0.391) ^ns^
	RobustScaler	0.6100	0.6100	0.6107	0.6104	0.326 (0.568) ^ns^
S5	None	0.7315	0.8434	0.7165	0.7748	0.939 (0.625) ^ns^
	Z-score	0.7222	0.8283	0.6667	0.7387	0.776 (0.678) ^ns^
	Min-Max normalization	0.7778	0.8430	0.6849	0.7558	0.278 (0.870) ^ns^
	Percentile	0.7037	0.8174	0.6593	0.7299	0.539 (0.764) ^ns^
	Maximum absolute scaling	0.7778	0.8430	0.6849	0.7558	0.278 (0.870) ^ns^
	RobustScaler	0.7685	0.8388	0.6591	0.7381	0.313 (0.855) ^ns^

Note: *, **, and ^ns^ represent significant differences at the 0.05 and 0.01 levels and non-significance, respectively

As an example from Table 3, D1 with raw data for *k-*means clustering compared with the true grouped data attained accuracy, precision, recall, and F-score values of 0.5614, 0.4949, 0.6058, and 0.5448 respectively. In comparison, feature scaling beforehand using Z-score provided 0.9620, 0.9578, 0.9512, and 0.9545, respectively, while Min-Max normalization provided 0.8538, 0.8360, 0.8395, and 0.8378, respectively, Percentile transformation provided 0.8889, 0.8973, 0.8693, and 0.8831, respectively, Maximum absolute scaling provided 0.9152, 0.8951, 0.8978, and 0.8965, respectively and RobustScaler provided 0.9327, 0.9333, 0.9297, and 0.9315, respectively.

These results obviously indicate that when features in the dataset have different units, feature scaling beforehand improves the *k-*means clustering performance. For testing the hypothesis for homogeneity between the true grouped data and results of *k-*means clustering using the raw data, the Chi-square value was 19.829 (p-value < 0.001), which signifies a significant difference). In comparison, the Chi-square values when using the five feature-scaling methods all show non-significant differences. The results showed the same trend for datasets D2–D5.

As an example from Table 4, S1 contains five variables: MCG, GVH, AAC, ALM1, and ALM2, all with the same unit (percentage). The accuracy, precision, recall, and F-score results for S1 using raw data for *k-*means clustering compared with the true grouped data were 0.9191, 0.9164, 0.9073, and 0.9119, respectively. In comparison, feature scaling using Z-score beforehand provided 0.9338, 0.9257, 0.927, and 0.9267, respectively, while Min-Max normalization provided 0.9228, 0.9187, 0.9122, and 0.9154, respectively, Percentile transformation provided 0.9375, 0.9240, 0.9338, and 0.9289, respectively, Maximum absolute scaling provided 0.9191, 0.9164, 0.9073, and 0.9119, respectively and RobustScaler provided 0.9228, 0.9218, 0.9223, and 0.9220, respectively. These results indicate that when features in the dataset have the same units, feature scaling may not be required before *k*-means clustering. The results for the other datasets having features with the same unit are the same trend.

In summary, for a dataset having features with different units, neglecting feature scaling before *k-*means clustering leads to noticeably poor performance whereas for a dataset having features with the same unit, feature scaling before *k-*means clustering did not affect the performance.

**Fig 5:**
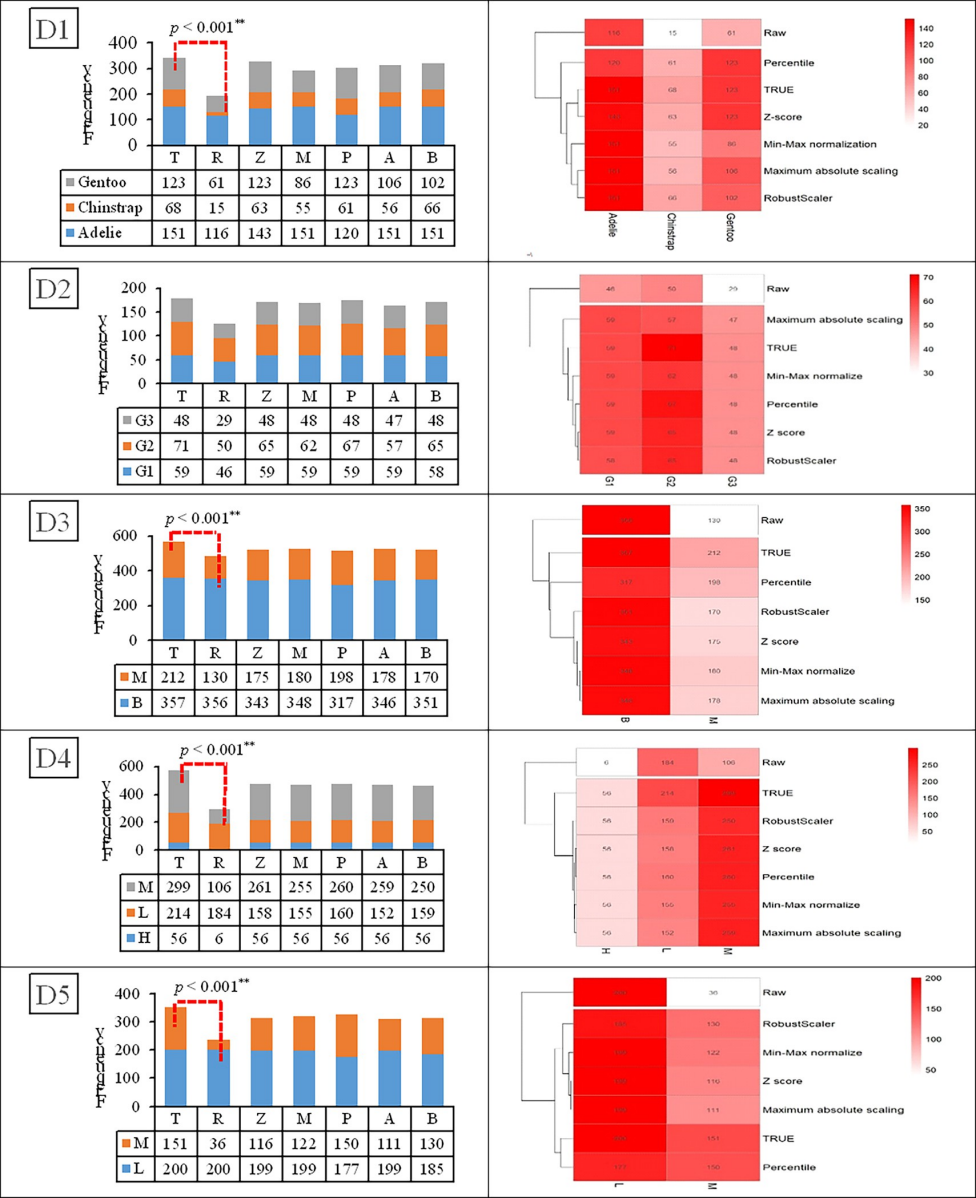
Stacked bar charts and heat maps for the datasets with different units (D1–D5). The dendrograms denote significant differences. R: raw data, Z: Z-score, M: Min-Max normalization, P: Percentile transformation, A: Maximum absolute scaling and B: RobustScaler.

**Fig 6:**
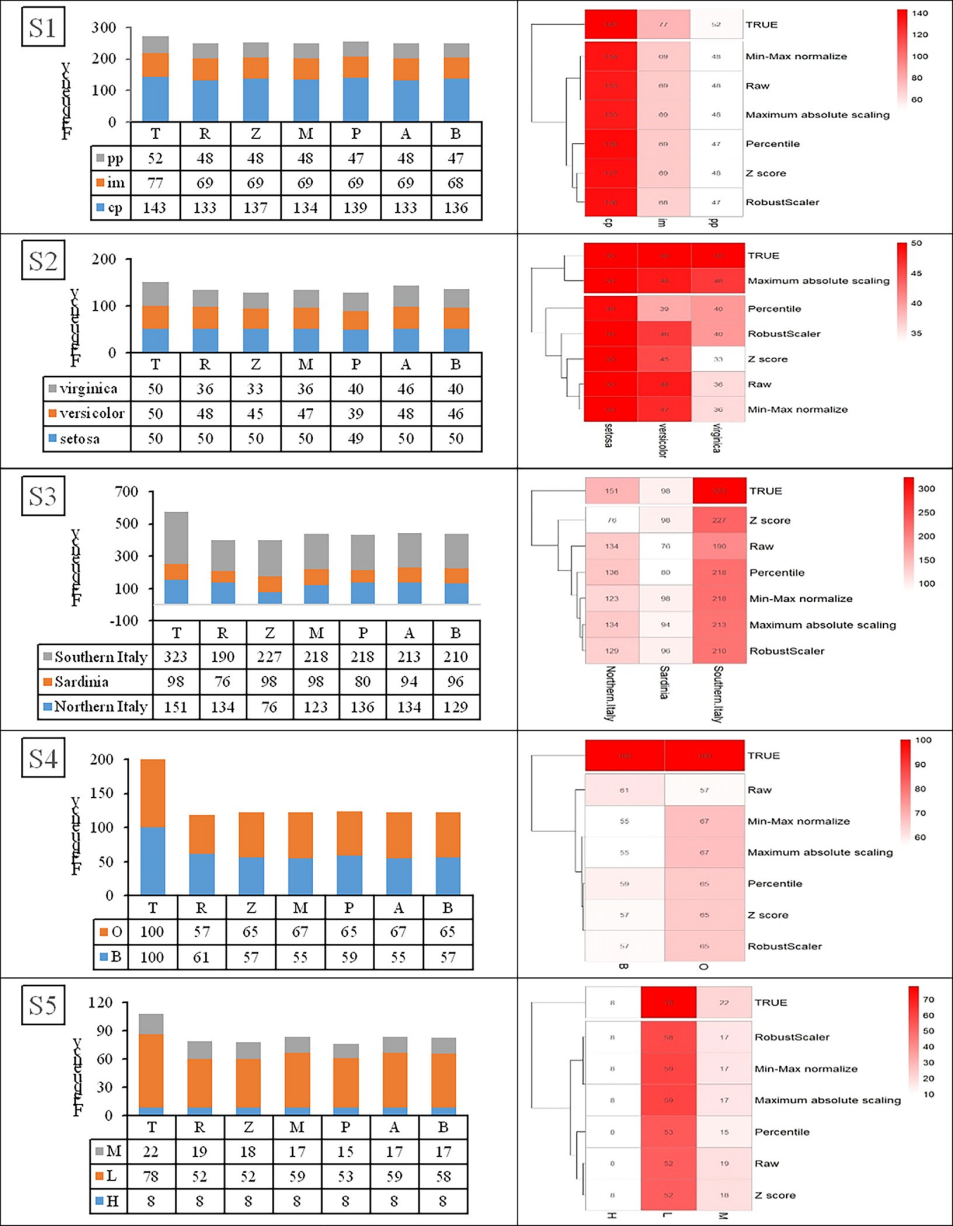
Stacked bar charts and heat maps for the datasets with the same units (S1–S5). R: raw data, Z: Z-score, M: Min-Max normalization, P: Percentile transformation, A: Maximum absolute scaling and B: RobustScaler.

To visualize the results for D1–D5 and S1–S5, stacked bar charts (left-hand side) and heatmaps (right-hand side) between the true grouped data and the results of *k-*means clustering are presented in Figs 5 and 6, respectively. For example, the stacked bar chart for D1 in Fig 5, shows the frequencies for three penguin species: gentoo, chinstrap, and Adélie of 123, 68, and 151, respectively. The results for *k-*means clustering using the raw data provide clusters of 61, 15, and 116, respectively, which when hypothesis for homogeneity testing with a significant difference (*p* < 0.001) leads to the conclusion that the true grouped data and *k-*means clustering results are different. However, the results of *k-*means clustering after applying a feature scaling method provided Gentoo, Chinstrap, and Adélie clusters of 123, 63, and 143, respectively, using Z-score; 86, 55, and 151, respectively, using Min-Max normalization; 123, 61, and 120, respectively, using Percentile transformation; and 105, 56, and 151, respectively, using Maximum absolute scaling; 102, 66, and 151, respectively, using RobustScaler. These are all close to the true grouped data and hypothesis for homogeneity testing for each provides the same conclusion of a non-significant difference between the true grouped data and *k*-means clustered data.

The results of the *k-*means clustering of D1–D5 are visualized using stacked bar charts and heatmaps in Fig 5, in which the Euclidean distance is used to test for similarity between the true grouped data and *k*-means clustered data. The dendrogram shows that the *k-*means clustered groups using the raw data are very different from those obtained using the feature scaling methods.

Similarly, Fig 6 shows stacked bar charts and heatmaps for S1–S5. For example, the true grouped data for S1 shows three *Escherichia coli* strains: pp, im, and cp with true frequencies of 52, 77, and 143, respectively. The *k-*means clustering results using raw data provided clusters of 48, 69, and 133, respectively. Meanwhile, Z-score provided clusters of 48, 69, and 137, respectively; Min-Max normalization provided clusters of 48, 69, and 134, respectively; Percentile transformation provided clusters of 47, 69, and 139, respectively; Maximum absolute scaling provided clusters of 48, 69, and 133, respectively and RobustScaler provided clusters of 47, 68, and 163, respectively. These are all close to the true grouped data, and hypothesis for homogeneity testing revealed only non-significant differences between the true groups and *k****-***means clusters in all cases. In addition, the dendrogram shows that the *k*-means clusters using the raw data are the same as when using the five scaling methods. The same trend in results was found for S2–S5.

The data visualization using stacked bar charts and heatmaps in Figs 5 and 6 indicates once again that feature scaling before *k-*means clustering analysis is essential for datasets in which the features have different units but not when they have the same unit.

**Fig 7:**
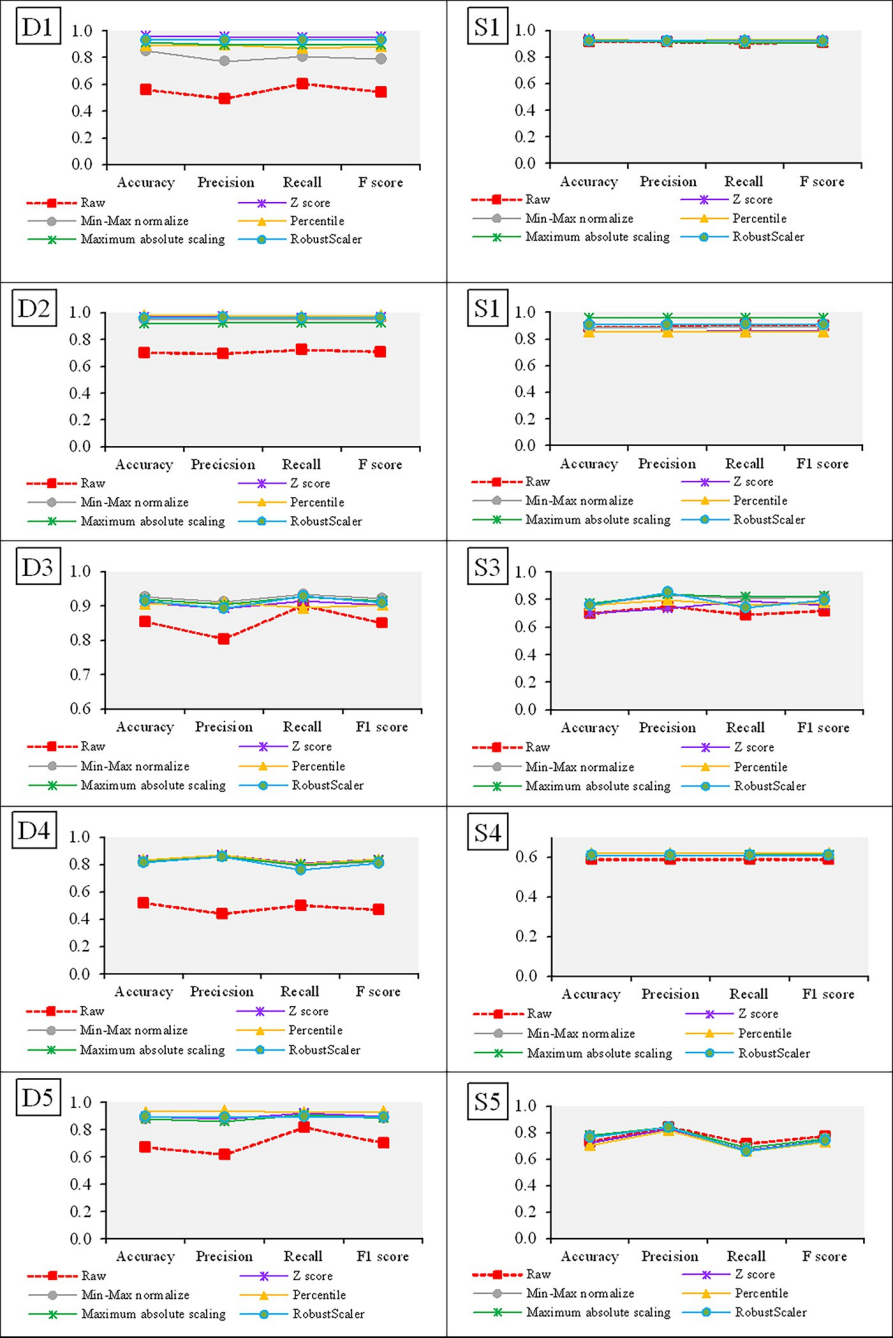
Plots of the performance metrics (accuracy, precision, recall, and F1-score) of the results of the *k-*means clustering of datasets with different (D1–D5) and the same (S1–S5) units.

To compare the performances of the various methods, plots of their accuracy, precision, recall, and F-scores for datasets having different or the same units are shown in Fig 7. For the datasets with different units, the performance metric results (accuracy, precision, recall, and F-score) using the five scaling methods are superior to when using the raw data whereas for the datasets with the same units, they provided similar results.

Table 5 provides the results for the overall average accuracy, precision, recall, and F-scores of *k*-means clustering for all of the datasets. For the datasets having features with different units, the Z-score method obtained accuracy, precision, recall, and F-score values of 0.9142, 0.9149, 0.9115, and 0.9128 respectively, which indicate its mostly superior performance to the other scaling methods. The only exception was the precision of the Percentile transformation method (0.9202). However, even the poor performance metrics for the Min-Max normalization were better than when using the raw data (accuracy, precision, recall, and F-score values of 0.8855, 0.8859, 0.8850, and 0.8851 versus 0.6621, 0.6112, 0.7104, and 0.6559, respectively). On the other hand, for datasets having features with the same unit, the Maximum absolute scaling method obtained accuracy, precision, recall, and F-score values of 0.8076, 0.8333, 0.7967, and 0.8131, respectively, which were slightly better than the other scaling methods and using raw data.

**Table 5:** Comparison of the average performance metric values for *k*-means clustering of datasets having features with different (D1–D5) or the same (S1–S5) units.

Method	D1–D5				S1–S5			
	Accuracy	Precision	Recall	F-Score	Accuracy	Precision	Recall	F-Score
R	0.6621	0.6112	0.7104	0.6559	0.7666	0.7987	0.7622	0.7792
Z	0.9142	0.9149	0.9115	0.9128	0.7641	0.7907	0.7714	0.7790
M	0.8855	0.8859	0.8850	0.8851	0.7930	0.8180	0.7828	0.7984
P	0.9079	0.9202	0.8949	0.9072	0.7746	0.8024	0.7650	0.7819
A	0.8911	0.8901	0.8909	0.8902	0.8076	0.8333	0.7967	0.8131
B	0.9047	0.9089	0.8958	0.9017	0.7937	0.8260	0.7693	0.7947

Note: Underlining indicated the best performance, R: raw data, Z: Z-score, M: Min-Max normalization, P: Percentile transformation, A: Maximum absolute scaling and B: RobustScaler.

**Fig 8:**
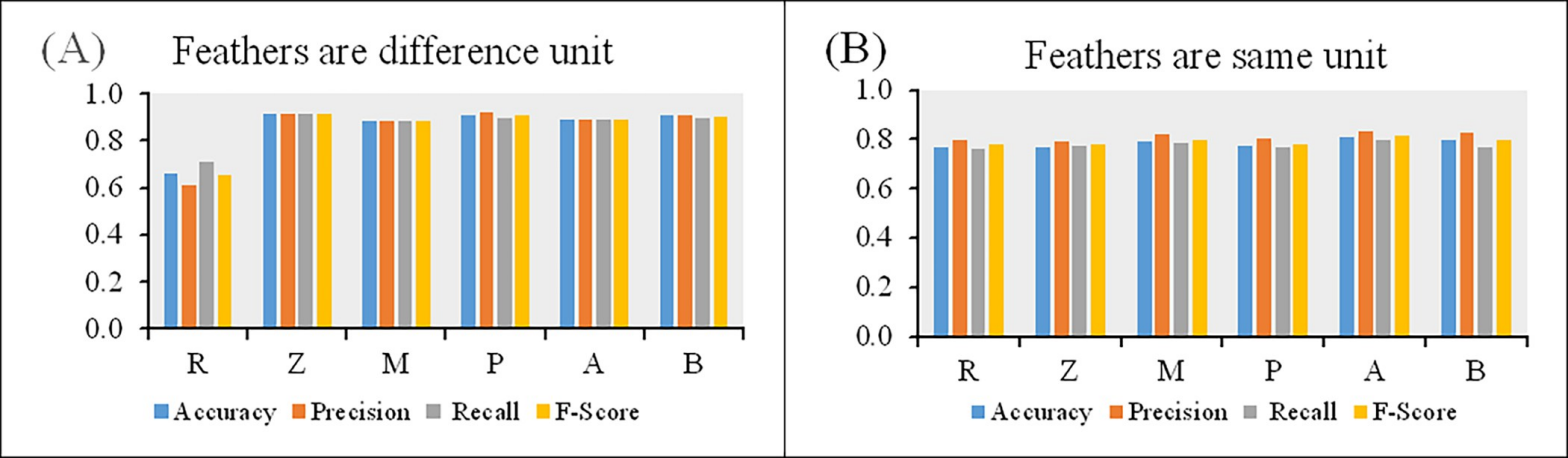
Comparison of the average performance metric values for *k*-means clustering of datasets having features with different (D1–D5) and the same (S1–S5) units. R: raw data, Z: Z-score, M: Min-Max normalization, P: Percentile transformation, A: Maximum absolute scaling and B: RobustScaler.

As shown in Fig 8(A) for the datasets having features with different scales, the results clearly demonstrate that when using the scaling methods before the *k-*means clustering analysis, the overall average performance metrics (accuracy and precision, recall, and F-score) were superior to using the raw data. In contrast, as shown in Fig 8(B) for the datasets having features in the same unit, the performance metrics results are similar, with feature scaling using Maximum absolute scaling being slightly better than using the other scaling methods or raw data.

## 6. Conclusions

Feature scaling, a crucial aspect of pre-processing, is particularly significant for distance-based algorithms like *k-*means clustering. This algorithm relies on feature scaling to ensure equal distance consideration across all features. The direct impact of distance-based scales on cluster assignment is a key concern. In particular, *k-*means clustering uses Euclidean distance between data points, so features with larger values or ranges will have a greater impact on the clustering results than features with smaller ones.

This study clarifies this problem and focuses on whether feature scaling should be used before *k*-means clustering analysis. To this end, the *k-*means clustering performance using raw data was compared with preprocessing the data using five feature-scaling techniques (Z-score, Min-Max normalization, Percentile transformation, Maximum absolute scaling and RobustScaler) when the true group membership is known. Ten real data sets, five with features having the same unit and five with features with different units, were used in the evaluation. Four performance measures, accuracy, precision, recall, and F-score, were used to compare the performances of the various techniques. The results indicate that the feature-scaling step is crucial and should not be neglected when performing *k*-means clustering on datasets containing features with different units, which leads to more accurate and reliable cluster assignments. The findings results are consistent with those from [39, 40]. Although feature scaling using Maximum absolute scaling is slightly better than the other scaling methods and raw data when the dataset contains features with the same unit, the improvement was not significant.

The main contribution of this study’s findings confirms that feature scaling, particularly when the features in the dataset have different units commonly found in real-world data sets, helps prevent features with larger magnitudes from dominating the distance calculations, leading to more accurate and consistent clustering results and better identification of the natural groupings in the data. Ignoring scale in *k*-means clustering can distort results as features with larger values dominate. Since Euclidean distance, the basis of *k*-means is sensitive to such variations. At the same time, the feature scaling in *k*-means clustering performed slightly better than the raw data when the dataset contained features with the same unit; the improvement was insignificant. This study’s findings indicate that feature scaling is crucial and should not be neglected when performing *k*-means clustering, especially on datasets with features having different units. According to recent research [21, 28], a high magnitude affects the distance between two given points, which impacts the performance of *k-*means clustering since variables with higher magnitudes are given more weight. Therefore, it is always advisable for researchers to bring all the features to the same scale before applying distance-based algorithms such as k-means clustering [22].

However, this study only employs a *k*-means clustering algorithm to determine the similarity between data points using Euclidean distance by focusing on the impact of feather scaling of datasets with the same units and different units. In the future, other algorithms can be compared using different distance measures to achieve better clustering performance.
